# Acceptability patterns of hypothetic taxes on different types of foods in France

**DOI:** 10.1017/S1368980024002556

**Published:** 2024-12-26

**Authors:** Florian Manneville, Barthélemy Sarda, Emmanuelle Kesse-Guyot, Sandrine Péneau, Bernard Srour, Julia Baudry, Benjamin Allès, Yann Le Bodo, Serge Hercberg, Mathilde Touvier, Chantal Julia

**Affiliations:** 1 Université Sorbonne Paris Nord and Université Paris Cité, INSERM, INRAE, CNAM, Center of Research in Epidemiology and StatisticS (CRESS), Nutritional Epidemiology Research Team (EREN), 74 rue Marcel Cachin, Bobigny F-93017, Cedex, France; 2 Public Health Department, Avicenne Hospital, Assistance Publique-Hôpitaux de Paris (AP-HP), Bobigny, France; 3 Department of Human and Social Sciences, EHESP School of Public Health, 15 avenue du Professeur Léon Bernard, CS 74312, Rennes F-35043, Cedex, France; 4 University of Rennes, Arènes Research Unit, UMR CNRS 6051, Rennes F-35000, France

**Keywords:** Food, Tax, Acceptability, Population, Latent class analysis

## Abstract

**Objective:**

To identify patterns of food taxes acceptability among French adults and to investigate population characteristics associated with them.

**Design:**

Cross-sectional data from the NutriNet-Santé e-cohort. Participants completed an *ad hoc* web-based questionnaire to test patterns of hypothetical food taxes acceptability (i.e. overall perception combined with reasons for supporting or not) on eight food types: fatty foods, salty foods, sugary foods, fatty and salty foods, fatty and sugary products, meat products, foods/beverages with unfavourable front-of-pack nutrition label and ‘ultra-processed foods’. Sociodemographic and anthropometric characteristics and dietary intakes (24-h records) were self-reported. Latent class analysis was used to identify patterns of food taxes acceptability.

**Setting:**

NutriNet-Santé prospective cohort study.

**Participants:**

Adults (*n* 27 900) engaged in the French NutriNet-Santé e-cohort.

**Results:**

The percentage of participants in favour of taxes ranged from 11·5 % for fatty products to 78·0 % for ultra-processed foods. Identified patterns were (1) ‘Support all food taxes’ (16·9 %), (2) ‘Support all but meat and fatty products taxes’ (28·9 %), (3) ‘Against all but UPF, Nutri-Score and salty products taxes’ (26·5 %), (4) ‘Against all food taxes’ (8·6 %) and (5) ‘No opinion’ (19·1 %). Pattern 4 had higher proportions of participants with low socio-economic status, BMI above 30 kg/m^2^ and who had consumption of foods targeted by the tax above the median.

**Conclusions:**

Results provide strategic information for policymakers responsible for designing food taxes and may help identify determinants of support for or opposition to food taxes in relation to individual or social characteristics or products taxed.

Chronic diseases such as cancer and CVD are the leading causes of death worldwide^(1)^. Dietary intake is known to be a modifiable risk factor for some chronic diseases with 11 million deaths and 255 million disability-adjusted life-years attributable to dietary risk factors^(2)^. Meta-analyses have shown that consumption of at least five servings of fruits and vegetables reduces the risk of cardiovascular death by 10–25 %^(3)^. Additionally, studies have shown an increased risk of developing cardiometabolic disease associated with higher consumption of ultra-processed foods (UPF)^(4)^, saturated fat^(5)^ and red and processed meat^(6)^.

The extant literature emphasises the need to improve the dietary intake of the population in order to prevent chronic diseases. Among existing strategies, food taxation could serve several public health purposes^(7)^. First, increasing the price of less healthy types of foods could discourage consumers from purchasing them. Second, the tax could raise revenue that could be reinvested to benefit the health of the population. Finally, the tax could encourage manufacturers to reformulate their products, especially if the tax rate varies according to the nutritional composition of the product. From a consumer perspective, in theory, a tax on certain types of foods would increase their price and create an incentive for consumers to avoid these foods^(8)^. This incentive is based on the concept of price elasticity of demand. Price elasticity is defined as the change in the quantity of food purchased in response to a change in the price of that food and differs for different types of food, with a higher elasticity for beef than for fats or sweets, for example^(9)^. The literature suggests that taxes on sugary, fatty and salty products^(10)^, sugar-sweetened beverages^(11)^, UPF^(12)^, meat^(13)^ and tax based on the Nutri-Score^(14)^ could lead to a decrease in the purchase and/or consumption of unhealthy foods and improve the health of the population.

In the context of declining revenues and increasing chronic diseases, food taxes are of increasing interest to governments^(15)^. Indeed, these taxes are sometimes seen as win-win policies, as they can improve health, reduce healthcare costs, mobilise revenue and contribute to reducing social inequalities in health and greenhouse gas emissions (for meat tax)^(15,16)^. In France, a flat tax on sweetened beverages was introduced in 2012 and was particularly well received in terms of its potential to improve the health of the population^(17)^. This tax led to a modest reduction (about 10 ml) in weekly per capita purchases of sweetened beverages^(18)^. It was revised in 2018 (indexing the amount of the tax to the sweetener content) and was still largely perceived positively by the population, in part because it was framed as a public health measure rather than a new source of revenue for the government^(19)^.

The usefulness of the tax on sweetened beverages tax in reducing their consumption has sparked interest among French policymakers to tax other types of foods, such as products with added sugars, high-fat products, meat, UPF and unfavourable Nutri-Score labelling (proposed by the French High Council of Public Health in 2017)^(20–22)^. This aligns with Eykelenboom *et al.* (2022) who concluded that targeting a broad range of food types could have a greater positive effect on consumer food purchases than taxing sweetened beverages alone^(14)^. However, an important policy consideration in introducing such food taxes for dietary change is the attitude of the population towards such food taxes and the extent to which it is likely to be accepted by the population^(20,23)^. Acceptability of food taxes could be defined as the extent to which the population subject to food taxes perceives them as appropriate, based on anticipated or experienced cognitive or emotional responses to food taxes^(23)^. Acceptability is important because if a food tax is implemented and accepted by the population, it may be more likely to receive sustained support from the government and therefore more likely to be effective in reducing the population’s consumption of the targeted foods. Also, if a tax is not accepted by the population, it is possible that the population will continue to buy the taxed foods despite the price increase by adjusting their budget^(24)^. In addition to the acceptability of food taxes, the reasons for the acceptability of such taxes are important for understanding the public’s perception of food taxes. These reasons could be related to health, economic, sustainability, environmental, ethical, political and other values. There may be different patterns of acceptability of food taxes across the population when acceptability and reasons for acceptability are considered together. For example, part of the population might support a food tax on meat for environmental and animal welfare reasons, while another (potentially larger) part of the population would not support the tax due to its rather favourable nutritional composition. Therefore, it seems important to identify and characterise the patterns of acceptability of and reasons for accepting different types of food taxes across the population in order to guide policy in the implementation of such taxes. The aim of this study was to identify different patterns of acceptability of hypothetical food taxes among French adults and to investigate the population characteristics associated with the identified patterns.

## Method

### Population

This study used cross-sectional data from the web-based NutriNet-Santé cohort, which was launched in 2009 and is still ongoing, which included French volunteers to French volunteers aged 15 years and older. The objectives of the NutriNet-Santé study are to investigate the relationship between nutrition and health outcomes and to examine the determinants of dietary patterns and nutritional status using a web-based approach. At baseline, participants were asked to complete a set of questionnaires to assess dietary intake (via three 24-h dietary records randomly distributed over a 2-week period) and sociodemographic and anthropometric characteristics. These characteristics were updated every 6–12 months during the follow-up. An *ad hoc* questionnaire on the acceptability and reasons for supporting or opposing food taxes was administered to NutriNet-Santé study participants aged 18 years and older from 8 March 2021 to 28 June 2021. The questionnaire for this study was the second part of a wider questionnaire, the first part of which asked participants about the acceptability of sugar taxation.

After excluding participants from the NutriNet-Santé study without data on food tax acceptability (*n* 443), the study sample consisted of 29 000 participants. This sample was weighted to improve its representativeness of the French adult population. Weights were calculated by sex using the CALMAR macro according to the 2016 national census (i.e. the most recent data available) of the National Institute of Statistics and Economic Studies reports on sex, age, education level, occupation, region of residence and marital status^(25)^. The study protocol was previously published and fully described in detail elsewhere^(26)^.

### Measurements

#### Food taxes acceptability

The questionnaire focused on eight hypothetical taxes on foods with high levels of nutrients of concern and/or high environmental impact and/or high media coverage. A working group of experts in nutrition, public health and epidemiology developed the items. The questionnaire was then tested internally with other members of the research team (engineers, technicians, other researchers not involved in its development) and then tested on the first 1000 volunteers to correct errors and misunderstandings. In particular, they could ask questions or report problems spontaneously by email if they wished. They could also provide a feedback report at the end of the questionnaire. The acceptability of each food tax was measured by the following statement: ‘*If a new tax were to be introduced on certain foods, would you be in favour of including the following foods?*’ The food groups were fatty products (i.e. added fats such as butter and cream), sugary products (e.g. breakfast cereals, sweets), salty products (e.g. salted snacks, ready-made meals), fatty and sugary products (e.g. cookies, cakes, pastries), fatty and salty products (e.g. aperitifs, processed meat, cheeses), meat (including poultry), UPF and products with an unfavourable Nutri-Score front-of-pack labelling (i.e. scores D or E). Ultra-processed foods are those that have undergone intense industrial physical, chemical or biological processes (e.g. hydrogenation, moulding, extruding, preprocessing by frying) and/or that contain industrial substances not usually found in domestic kitchens (e.g. maltodextrin, hydrogenated oils or modified starches), cosmetic additives (e.g. dyes, emulsifiers, artificial sweeteners) or flavouring agents^(27)^. Nutri-Score is a summary front-of-pack nutrition label that uses five colours and letters (from green/A to dark orange/E) to help understand and compare the nutritional value of foods and has been voluntarily implemented in France since 2017^(28)^. Response options included ‘strongly disagree’, ‘somewhat disagree’, ‘neither agree nor disagree’, ‘somewhat disagree’ and ‘strongly agree’. Participants who responded ‘strongly agree’ or ‘somewhat agree’ were then asked to give up to two reasons for their support, from ‘low nutritional quality’, ‘unhealthy’, ‘mainly imported’, ‘high resource consumption’ (in terms of land or water use for production for instance), ‘high pesticide use’, ‘natural resources and biodiversity damage’, ‘unethical production’, ‘price not in line with its real value’ and ‘other’ (open response). Participants who answered ‘strongly disagree’ or ‘somewhat disagree’ were then asked to give up to two reasons for their opposition among ‘healthy’, ‘good nutritional quality’, ‘mainly produced in France’, ‘respect for the environment’, ‘price must not increase’, ‘concern over the fact that it would cover products that are too different’ (e.g. many different products would be subject to a tax on products with an unfavourable Nutri-Score label (e.g. D or E)), ‘infringement of consumer freedom of choice’, ‘not effective in reducing consumption’, ‘pretext to pay off public debt’ and ‘other’ (open answer).

#### Participant characteristics

Individual data reported by participants from the study period closest to the tax-related questionnaire (up to 2 years before or 1 year after the data collection for dietary intake) were used. Sociodemographic characteristics included sex (i.e. biological attribute [male/female]), age (years) categorised into five classes (18–30, 31–44, 45–54, 55–65, over 65), household income per month per consumption unit^(29)^ (CU) (€) categorised into four classes (less than €1300/month/CU, €1300–2600/month/CU, over €2600/month/CU, do not want to declare), education level categorised into three classes (no high school diploma, high school diploma, university degree), occupation, region of residence, marital status (married, in a couple, divorced/separated, widowed, single), number of persons in the household, child aged 0–13 years old in the household (yes/no) and adolescent aged 14–18 years old in the household (yes/no). The anthropometric characteristic was BMI (kg/m^2^) based on self-reported height and weight and categorised into four classes (under 18·5 kg/m², 18·5–25 kg/m², 25–30 kg/m², over 30 kg/m²). As dietary intake data were not available for all participants, BMI was used as a proxy for dietary intake. Average daily intakes (g/d) of fatty products, sugary products, salty products, fatty and sugary products, fatty and salty products, meat, products with unfavourable Nutri-Score and UPF were assessed using the 24-h dietary records. Daily consumption was then categorised into two classes for each food group based on the corresponding median consumption in the sample (≤ median, > median).

### Statistical analyses

For each food tax, we derived nineteen dichotomous (yes/no) variables combining food tax acceptability and reasons in favour and against the food tax. All dichotomous variables are presented on the abscissa axis in online supplementary material, Supplemental Fig. S1. Responses from participants who neither agreed nor disagreed with a food tax were coded as ‘no’, as they were not asked to give the reasons for being in favour or against the food tax.

#### Descriptive analyses

We computed numbers and percentages for all variables (participant characteristics, acceptability of food taxes, reasons in favour or against food taxes). Medians (Q1–Q3) were described for participants’ dietary intake.

#### Identification of patterns of food taxes acceptability

Latent class analysis was used to derive patterns of acceptability and reasons for acceptability of the eight hypothetical food taxes^(30)^. This method allows the identification of unobserved (i.e. latent) homogeneous patterns in a heterogeneous population using a process that considers an incremental number of classes (i.e. patterns). The selection of the optimal number of patterns was based on minimising the Akaike information criteria, the Bayesian information criteria (closest to 0), the posterior probabilities (> 80 %), the proportion of participants in each pattern (> 5 %) and the interpretation of the pattern^(30)^. Entropy (range 0–1), which represents the probability of each participant being in each pattern, was also computed to indicate how accurately the model defined the patterns^(30)^. Latent class analysis determines item-response probabilities (i.e. the probability that participants will select a particular response) conditional on pattern membership. Comparing item-response probabilities across patterns allows each identified pattern to be distinguished^(30)^. Cross-tabulations were used to describe the distribution (%) of food taxes acceptability according to identified patterns.

#### Associations between participants’ characteristics and patterns of food taxes acceptability

Adjusted and unadjusted multinomial logistic regressions were used to model the associations between sociodemographic characteristics, anthropometric characteristics and dietary intake and patterns of food taxes acceptability. The most common pattern was used as a reference. OR, 95 % CI and adjusted column percentages are reported.

Data were analysed using SAS v9.4 (SAS Institute Inc.) and PROC latent class analysis. Figures were generated using Microsoft Excel.

## Results

### Participants’ characteristics

The crude (i.e. before weighting) sample was composed of 74·5 % females, 28·0 % participants aged over 65 years, 33·8 % participants with a household income over €2600/month/CU and 71·3 % participants with a university degree (Table 1). After weighting, 52·4 % of the participants were female, 21·7 % were aged 31–44 years old, 43·0 % lived in a household with an income of 1300–1600€/month/CU and 42·9 % had a high school diploma. A total of 55·1 % of participants had a BMI between 18·5 and 25 kg/m². The median daily consumption of UPF, products with an unfavourable Nutri-Score labelling and salty products were 346·8 (259·4–459·4) g/d, 197·2 (142·5–280·1) g/d and 140·9 (87·2–201·6) g/d, respectively (see online supplementary material, Supplemental Table S1).

**Table 1.** Description of participants’ sociodemographic and anthropometric characteristics, crude and after weighting (*n* 27 900)

	Crude		Weighted
	*n*	%	%
**Sociodemographic characteristics**			
Sex			
Male	13 289	25·5	47·6
Female	14 611	74·5	52·4
Age			
18–30	4293	4·2	15·4
31–44	6062	19·5	21·7
45–54	8146	21·4	29·2
55–65	4584	26·9	16·4
Over 65	4815	28·0	17·3
Household income			
Less than €1300/month/CU	6392	11·0	22·9
€1300–2600/month/CU	12 007	40·7	43·0
Over €2600/month/CU	5648	33·8	20·2
Do not want to declare	3853	14·4	13·8
Educational level			
No high school diploma	2857	1·9	10·2
High school diploma	11 979	26·9	42·9
University degree	13 064	71·3	46·8
Occupation			
Farmer	268	0·2	1·0
Artisan, shopkeeper, business owner	1086	2·3	3·9
Manager of higher intellectual profession	3600	22·1	12·9
Intermediate profession	2916	9·5	10·4
Employee	5036	10·9	18·0
Worker	2918	0·5	10·5
Student	1928	1·4	6·9
Retired person	4237	47·2	15·2
No activity	5911	5·8	21·2
Region of residence			
North	2538	3·5	9·1
Ⓘle-de-France	5074	19·7	18·2
Paris basin	2627	14·3	9·4
East Center	3272	13·8	11·7
East	3437	7·8	12·3
West	3087	15·2	11·1
South West	5407	12·6	19·4
Mediterranean	2458	13·1	8·8
Marital status			
Married	12 874	55·3	46·1
In a couple	5859	17·1	21·0
Divorced/separated	2349	11·0	8·4
Widowed	851	4·7	3·1
Single	5967	12·0	21·4
Number of persons in the household			
1	6953	22·3	24·9
2	11 350	50·1	40·7
3	4725	12·1	16·9
4	3283	11·2	11·8
5	1589	4·2	5·7
Child aged 0–13 years old in the household			
No	21 199	78·8	76·0
Yes	6701	21·2	24·0
Adolescent aged 14–18 years old in the household			
No	25 051	91·7	89·8
Yes	2849	8·3	10·2
**Anthropometric characteristic**			
BMI			
Under 18·5 kg/m²	1330	4·8	4·8
18·5–25 kg/m²	15 362	59·2	55·1
25–30 kg/m²	7452	25·1	26·7
Over 30 kg/m²	3756	10·9	13·5

### Acceptability of food taxes

**Figure 1.**
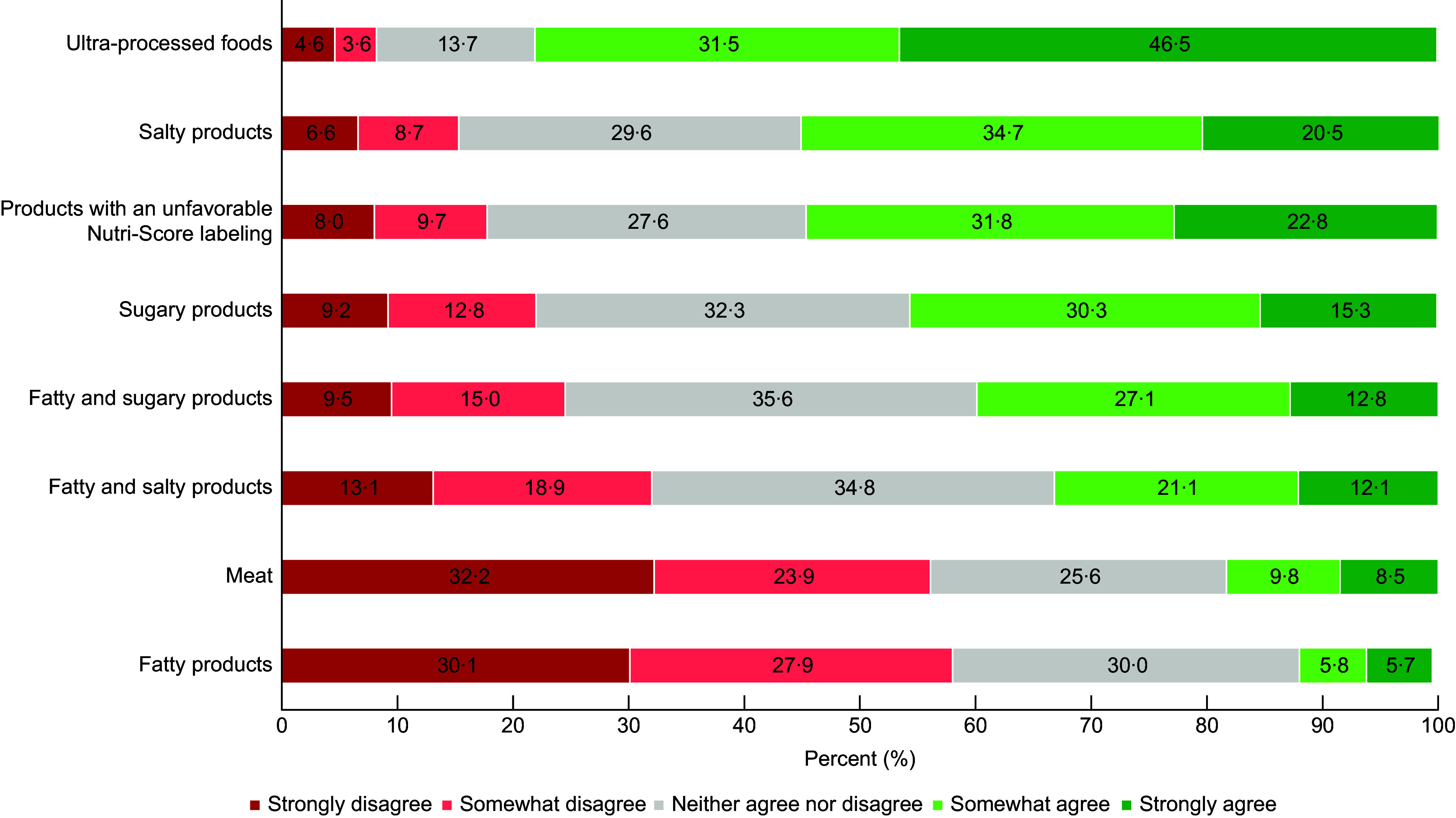
Description of taxes acceptability among all participants (weighted sample) (*n* 27 900).

Taxes on UPF, salty products and products with an unfavourable Nutri-Score labelling were the most commonly accepted (Fig. 1). For example, 78·0 % of participants strongly or somewhat agreed with the tax on UPF. Taxes on fatty products and meat were the least accepted. A total of 18·3 and 11·5 % of the participants agreed (strongly or somewhat) with taxes on fatty products and meat, respectively. The proposed UPF tax had the lowest level of ambivalence or uncertainty towards the tax; 86·3 % of participants agreed or disagreed with the UPF tax and only 13·7 % neither agreed nor disagreed.

### Reasons in favour and against food taxes

The distribution of reasons in favour and against food taxes is in Table 2. Most of the participants indicated ‘low nutritional quality’ and ‘unhealthy’ as reasons in favour of all taxes except those of fatty products and meat. ‘Unhealthy’ and ‘high resource consumption’ were the most common reasons for supporting taxes on fatty products and meat, respectively. Reasons against the most accepted tax (i.e. UPF tax) were mainly ‘not effective in reducing consumption’, ‘infringement of consumer freedom of choice and ‘pretext to pay off public debt’. For the least accepted taxes (i.e. fatty products and meat taxes), ‘good nutritional quality’ was the most frequently cited reason for disagreement.

**Table 2.** Description of reasons in favour of food taxes and against food taxes among participants (subsample strongly agree or somewhat agree)

	Total (*n* %)															
	Fatty products (e.g. butter, cream, etc.)		Sugary products (e.g. breakfast cereals, sweets, etc.)		Salty products (e.g. ready-made meals, salted snacks, etc.)		Fatty and sugary products (e.g. cookies, cakes, pastries, etc.)		Fatty and salty products (e.g. aperitifs, cold meat, cheeses, etc.)		Meat (including poultry)		Products with an unfavourable Nutri-Score labelling (e.g. D or E)		Ultra-processed foods	
	*n*	%	*n*	%	*n*	%	*n*	%	*n*	%	*n*	%	*n*	%	*n*	%
Reasons in favour of food taxes (subsample strongly agree or somewhat agree)																
Total	3228		12 732		15 395		11 226		9267		5097		15 257		21 774	
Low nutritional quality	816	25·3	9583	75·3	11 606	75·4	8270	74·3	5963	64·3	305	6·0	12 126	79·5	16 427	75·4
Unhealthy	1679	52·0	10 942	85·9	12 784	83·0	9388	84·4	7662	82·7	1019	20·0	12 493	81·9	17 232	79·1
Mainly imported	52	1·6	355	2·8	307	2·0	186	1·7	135	1·5	321	6·3	388	2·5	870	4·0
High resource consumption	999	30·9	492	3·9	541	3·5	416	3·7	796	8·6	2947	57·8	365	2·4	1308	6·0
High pesticide use	92	2·8	240	1·9	362	2·4	285	2·6	183	2·0	370	7·3	252	1·7	426	2·0
Natural resources and biodiversity damage	784	24·3	593	4·7	621	4·0	487	4·4	813	8·8	2172	42·6	633	4·1	1652	7·6
Unethical production	1048	32·5	337	2·6	580	3·8	246	2·2	780	8·4	1848	36·3	446	2·9	1360	6·2
Price not in line with its real value	167	5·2	716	5·6	1253	8·1	705	6·3	475	5·1	399	7·8	924	6·1	1449	6·7
Other	110	3·4	71	0·6	90	0·6	107	1·0	64	0·7	184	3·6	190	1·2	87	0·4
Reasons against food taxes (subsample strongly disagree or somewhat disagree)																
Total	16 308		6158		4256		6834		8909		15 656		4951		2294	
Healthy	2625	16·1	230	3·7	93	2·2	119	1·7	376	4·2	4474	28·6	119	2·4	120	5·2
Good nutritional quality	8900	54·6	1461	23·7	664	15·6	1376	20·1	3035	34·1	9622	61·5	885	17·9	209	9·1
Mainly produced in France	4339	26·6	165	2·7	125	2·9	562	8·2	1741	19·5	2945	18·8	39	0·8	16	0·7
Respect for the environment	168	1·0	33	0·5	20	0·5	16	0·2	68	0·8	188	1·2	19	0·4	13	0·6
Price must not increase	3815	23·4	1278	20·7	1080	25·4	1700	24·9	1602	18·0	3551	22·7	727	14·7	249	10·8
Concern products that are too different	1091	6·7	985	16·0	737	17·3	1396	20·4	2277	25·6	1077	6·9	1380	27·9	230	10·0
Infringement of consumer freedom of choice	2515	15·4	2005	32·6	1340	31·5	2353	34·4	2437	27·3	2789	17·8	1639	33·1	890	38·8
Not effective in reducing consumption	2520	15·5	2305	37·4	1356	31·9	2325	34·0	2013	22·6	1454	9·3	1425	28·8	1034	45·1
Pretext to pay off public debt	1453	8·9	1252	20·3	884	20·8	1235	18·1	1104	12·4	1298	8·3	851	17·2	607	26·5
Other	725	4·4	339	5·5	269	6·3	331	4·8	388	4·4	571	3·6	603	12·2	199	8·7

### Identification of patterns of food taxes acceptability

**Figure 2.**
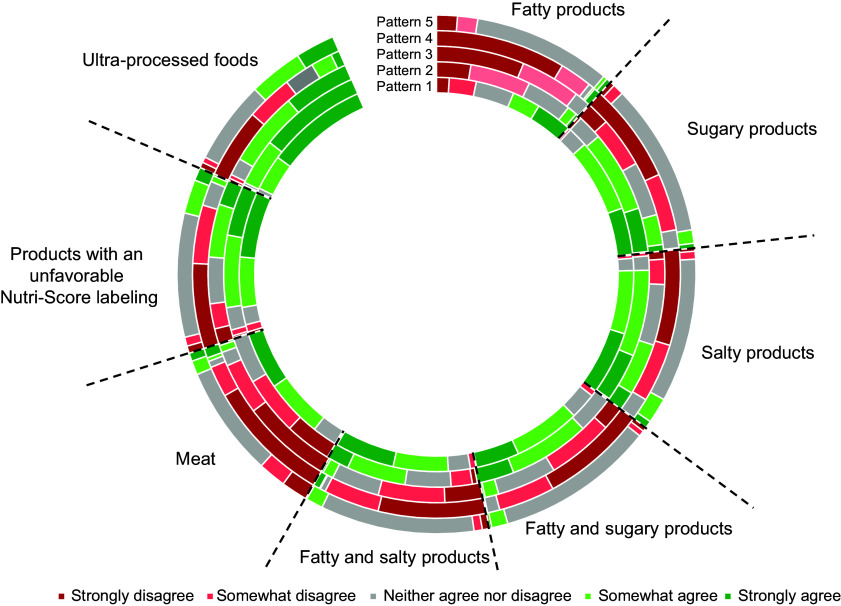
Graphical representation of the distribution acceptability for food taxes according to identified patterns of food taxes acceptability (*n* 27 900). Pattern 1, ‘Support all food taxes’; Pattern 2, ‘Support all but meat and fatty products taxes’; Pattern 3, ‘Against all but UPF, Nutri-Score and salty products taxes’; Pattern 4, ‘Against all food taxes’; Pattern 5, ‘No opinion’. Note: Patterns of food taxes acceptability were identified using latent class analysis. Figure 2 is the graphical representation of the numerical data presented in the online supplementary material, Supplemental Table S3. For each food tax, the proportions of participants who ‘strongly disagree’, ‘somewhat disagree’, ‘neither agree nor disagree’, ‘strongly disagree’ or ‘somewhat disagree’ in each pattern are shown. For example, for the tax on fatty products, in Pattern 1, 8·9 % of participants ‘strongly disagree’ (dark red zone), 18·9 % ‘somewhat disagree’ (light red zone), 27·0 % ‘neither agree nor disagree’ (grey zone), 19·6 % ‘somewhat agree’ (light green zone) and 25·6 % ‘strongly agree’ (dark green zone).

Statistical criteria (i.e. Akaike information criteria, Bayesian information criteria, pattern size and posterior probabilities) were in the appropriate range for all models (see online supplementary material, Supplemental Table S2). Therefore, we elected a model with five patterns based on interpretability criteria. Identified patterns are presented in online supplementary material, Supplemental Fig. S1. The distribution of acceptability of food taxes according to the identified patterns is shown in Fig. 2 and online supplementary material, Supplemental Table S3. The patterns were labelled as follows:

- **Pattern 1 (*n* 4728, 16·9 %),** labelled **‘*Support all food taxes*’**: Participants in this pattern supported taxes on meat and fatty products for ‘high resource consumption’, ‘unhealthy’, ‘natural resources and biodiversity damage’ and ‘unethical production’ reasons and supported all other taxes for ‘low nutritional quality’ and ‘unhealthy’ reasons.
- **Pattern 2 (*****n*** **8050, 28·9 %),** labelled **‘*Support all but meat and fatty products taxes*’**. Participants in this pattern were against taxes on meat and fatty products for ‘good nutritional quality’ and ‘healthy’ reasons and supported all other taxes for ‘low nutritional quality’ and ‘unhealthy’ reasons’.
- **Pattern 3 (*n* 7384, 26·5 %),** labelled **‘*Against all but UPF, Nutri-Score and salty products taxes*’**, included participants against taxes on fatty products, meat, fatty and salty products and fatty and sugary products for ‘good nutritional quality’, ‘price must not increase’, ‘concern products that are too different’, ‘infringement of consumer freedom of choice’ and ‘inefficacity in reducing consumption’ reasons. This pattern included participants in favour of taxes on UPF and products with an unfavourable Nutri-Score labelling for ‘low nutritional quality’ and ‘unhealthy’ reasons.
- **Pattern 4 (*n* 2405, 8·6 %),** labelled **‘*Against all food taxes*’**. Participants in this pattern were against all taxes for ‘price must not increase’, ‘infringement of consumer freedom of choice’, ‘pretext to pay off public debt’ and ‘inefficacity in reducing consumption’ reasons. A potential tax on UPF was slightly more accepted relatively to other taxes, for ‘low nutritional quality’ and ‘unhealthy’ reasons.
- **Pattern 5 (*n* 5333, 19·1 %),** labelled **‘*No opinion*’**. This pattern included participants who mostly neither agreed nor disagreed with all food taxes.

### Associations between participants’ characteristics and patterns of food taxes acceptability

Girl participants were more likely to be in the ‘*Against all food taxes*’ (57·3 %) pattern than in the ‘*Support all but meat and fatty products taxes*’ (52·4 %) pattern (Table 4). Participants aged 18–30 years were more likely to be in the ‘*Against all food taxes*’ (21·4 %) pattern than in the ‘*Support all but meat and fatty products taxes*’ pattern (11·9 %). Participants with no high school diploma were more likely to be in the ‘*Against all food taxes*’ (13·0 %) and ‘*No opinion*’ (12·9 %) patterns than in the ‘Support all but meat and fatty products taxes’ (7·9 %). Participants with a child in the household were more likely to be in the ‘*Against all food taxes*’ pattern (43·4 %) than in the ‘*Support all but meat and fatty products taxes*’ (15·9 %). Participants with BMI greater than 30 kg/m² were more likely to be in the ‘*No opinion*’ (18·5 %) and ‘*Against all food taxes*’ (17·5 %) patterns than in the ‘*Support all but meat and fatty products taxes*’ (9·6 %). Participants consuming products with an unfavourable Nutri-Score labelling above the median were more likely to be in the ‘*Against all food taxes*’ (52·3 %) pattern than in the ‘*Support all but meat and fatty products taxes*’ (48·3 %) (Table 4). Participants consuming meat above the median were less likely to be in the ‘*Support all food taxes*’ pattern (32·3) than in the ‘*Support all but meat and fatty products taxes*’ (53·1 %). The participants consuming UPF above the median were more likely to be in the ‘*Against all food taxes*’ pattern (56·0 %) than in the ‘*Support all but meat and fatty products taxes*’ (46·4 %). Unadjusted results were similar (see online supplementary material, Supplemental Tables S4 and S5).

**Table 3.** Associations between participants’ sociodemographic and anthropometric characteristics and identified patterns: adjusted analyses (weighted sample) (*n* 27 900)

	Pattern 1			Pattern 2			Pattern 3			Pattern 4			Pattern 5		
	Support all food taxes			Support all but meat and fatty products taxes			Against all but UPF, Nutri-Score and salty products taxes			Against all food taxes			No opinion		
	*n* 4728, 16·9 %			*n* 8050, 28·9 %			*n* 7384, 26·5 %			*n* 2405, 8·6 %			*n* 5333, 19·1 %		
	OR*	95 % CI	%^†^	OR*	95 % CI	%^†^	OR*	95 % CI	%^†^	OR*	95 % CI	%^†^	OR*	95 % CI	%^†^
**Sociodemographic characteristics**															
Sex															
Male	Ref		50·7	Ref		47·6	Ref		43·9	Ref		42·7	Ref		51·1
Female	**0·9**	**0·8, 1·0**	**49·3**	Ref		52·4	**1·2**	**1·1, 1·2**	**56·1**	**1·2**	**1·1, 1·4**	**57·3**	**0·9**	**0·8, 0·9**	**48·9**
Age															
18–30 years	**1·5**	**1·3, 1·7**	**17·0**	Ref		11·9	**1·3**	**1·1, 1·5**	**15·9**	**1·7**	**1·4, 2·0**	**21·4**	**1·2**	**1·1, 1·4**	**16·0**
30–44 years	Ref		20·0			21·2	Ref		21·9	Ref		22·4	Ref		22·6
45–54 years	**1·3**	**1·1, 1·5**	**31·5**	Ref		25·8	1·1	1·0, 1·2	29·3	**1·2**	**1·0, 1·4**	**32·4**	1·1	1·0, 1·2	30·4
55–65 years	0·9	0·7, 1·0	14·5	Ref		17·7	1·0	0·9, 1·2	18·7	**0·7**	**0·6, 0·8**	**12·9**	**0·8**	**0·7, 0·9**	**14·7**
Over 65 years	**0·8**	**0·6, 1·0**	**17·0**	Ref		23·4	**0·6**	**0·5, 0·8**	**14·1**	**0·4**	**0·3, 0·6**	**10·9**	**0·6**	**0·5, 0·8**	**16·3**
Income															
Less than €1300/month/CU	1·0	0·9, 1·1	29·2	Ref		26·7	**0·7**	**0·6, 0·7**	**22·5**	**0·6**	**0·5, 0·6**	**19·0**	**0·6**	**0·5, 0·6**	**18·1**
€1300–2600/month/CU	Ref		41·8	Ref		37·8	Ref		46·3	Ref		43·8	Ref		44·8
Over €2600/month/CU	**0·7**	**0·6, 0·8**	**18·7**	Ref		23·6	**0·6**	**0·6, 0·7**	**18·5**	**0·8**	**0·7, 0·9**	**21·2**	**0·7**	**0·6, 0·8**	**19·4**
Did not want to declare	**0·8**	**0·7, 0·9**	**10·3**	Ref		11·8	0·9	0·8, 1·0	12·6	**1·2**	**1·1, 1·3**	**16·0**	**1·3**	**1·1, 1·4**	**17·7**
Education															
No high school diploma	1·1	0·9, 1·3	8·6	Ref		7·9	1·1	1·0, 1·2	10·0	**1·5**	**1·3, 1·8**	**13·0**	**1·5**	**1·3, 1·7**	**12·9**
High school diploma	Ref		39·4			40·4	Ref		46·5	Ref		43·2	Ref		43·5
University degree	1·0	0·9, 1·1	52·1	Ref		51·6	**0·7**	**0·7, 0·8**	**43·5**	**0·8**	**0·7, 0·9**	**43·8**	**0·8**	**0·7, 0·9**	**43·6**
Occupation															
Farmer	**0·1**	**0·0, 0·3**	**0·1**	Ref		0·8	**2·2**	**1·6, 2·9**	**1·7**	**0·3**	**0·1, 0·9**	**0·2**	**2·1**	**1·4, 3·1**	**1·1**
Artisan, shopkeeper, business owner	1·1	0·8, 1·3	3·8	Ref		3·3	1·1	0·9, 1·3	3·7	**1·4**	**1·0, 1·8**	**3·5**	**2·1**	**1·7, 2·6**	**4·8**
Manager of higher intellectual profession	Ref		15·1	Ref		14·2	Ref		14·4	Ref		10·9	Ref		9·8
Intermediate profession	**0·6**	**0·5, 0·7**	**9·8**	Ref		15·9	0·6	0·6, 0·7	10·3	0·9	0·7, 1·1	11·1	**0·5**	**0·4, 0·6**	**5·7**
Employee	**0·7**	**0·6, 0·8**	**14·1**	Ref		18·4	0·9	0·8, 1·1	17·7	**1·6**	**1·3, 1·9**	**22·5**	**1·5**	**1·3, 1·7**	**18·9**
Worker	0·8	0·7, 1·0	10·8	Ref		12·0	**0·7**	**0·6, 0·8**	**8·4**	**0·5**	**0·4, 0·7**	**4·7**	**1·5**	**1·3, 1·8**	**12·4**
Student	1·1	1·0, 1·4	8·3	Ref		6·7	**0·7**	**0·6, 0·8**	**4·7**	0·9	0·7, 1·3	4·9	**1·9**	**1·6, 2·3**	**8·9**
Retired person	**1·5**	**1·2, 1·9**	**15·9**	Ref		9·6	**1·8**	**1·4, 2·2**	**17·5**	**1·8**	**1·3, 2·5**	**13·4**	**2·8**	**2·2, 3·5**	**18·3**
No activity	1·1	1·0, 1·2	22·1	Ref		19·2	1·1	1·0, 1·3	21·6	**1·9**	**1·6, 2·3**	**28·7**	**1·5**	**1·3, 1·8**	**20·0**
Region of residence															
North	1·0	0·9, 1·2	10·4	Ref		9·1	1·1	1·0, 1·3	10·8	**0·8**	**0·6, 1·0**	**8·2**	0·9	0·8, 1·0	7·2
Ⓘle-de-France	Ref		20·1	Ref		18·1	Ref		19·0	Ref		20·7	Ref		16·1
Paris basin	**0·6**	**0·5, 0·7**	**7·0**	Ref		9·9	1·0	0·8, 1·1	10·0	**0·8**	**0·7, 1·0**	**9·3**	1·1	0·9, 1·2	9·4
East Center	**0·7**	**0·6, 0·8**	**9·1**	Ref		11·8	1·0	0·9, 1·1	12·7	1·1	0·9, 1·3	14·8	1·1	0·9, 1·2	11·1
East	1·0	0·8, 1·1	14·4	Ref		13·6	**0·8**	**0·7, 0·9**	**11·9**	**0·6**	**0·5, 0·7**	**8·8**	0·9	0·8, 1·1	11·5
West	**0·8**	**0·7, 0·9**	**9·4**	Ref		10·8	1·0	0·9, 1·2	11·7	0·9	0·7, 1·1	10·9	**1·2**	**1·0, 1·4**	**11·5**
South West	1·1	1·0, 1·2	22·3	Ref		19·5	**0·8**	**0·7, 0·9**	**16·8**	0·9	0·8, 1·0	20·0	**1·2**	**1·0, 1·3**	**20·3**
Mediterranean	0·9	0·8, 1·1	7·2	Ref		7·1	1·0	0·8, 1·1	7·2	0·9	0·7, 1·1	7·4	**2·0**	**1·7, 2·3**	**12·8**
Marital status															
Married	Ref		37·5	Ref		52·9	Ref		47·7	Ref		44·5	Ref		42·0
In a couple	**1·7**	**1·5, 1·9**	**22·4**	Ref		18·7	**1·3**	**1·2, 1·5**	**22·4**	**1·4**	**1·2, 1·6**	**22·0**	**1·4**	**1·3, 1·7**	**20·9**
Divorced or separated	**1·8**	**1·6, 2·1**	**10·8**	Ref		8·3	1·0	0·8, 1·1	7·4	**1·3**	**1·1, 1·6**	**9·4**	**1·3**	**1·1, 1·5**	**8·3**
Widow	0·9	0·7, 1·2	2·2	Ref		3·4	0·8	0·7, 1·0	2·6	0·7	0·5, 1·1	2·2	**1·3**	**1·1, 1·7**	**3·7**
Single	**2·3**	**2·0, 2·7**	**27·0**	Ref		16·6	**1·3**	**1·2, 1·5**	**19·8**	**1·6**	**1·3, 1·9**	**22·0**	**1·9**	**1·6, 2·2**	**25·1**
Number of persons in the household															
1	**0·6**	**0·5, 0·7**	**20·7**	Ref		23·6	1·0	0·8, 1·1	25·3	0·9	0·8, 1·1	29·2	**1·1**	**1·0, 1·3**	26·3
2	Ref		51·4	Ref		36·8	Ref		41·0	Ref		50·7	Ref		36·0
3	**0·5**	**0·4, 0·5**	**12·6**	Ref		19·7	**0·8**	**0·7, 0·8**	**16·7**	**0·4**	**0·3, 0·5**	**10·7**	1·0	0·8, 1·1	18·5
4	**0·4**	**0·4, 0·5**	**8·2**	Ref		13·8	**0·8**	**0·7, 0·9**	**12·4**	**0·3**	**0·3, 0·5**	**6·3**	0·9	0·8, 1·1	12·5
>=5	0·9	0·7, 1·0	7·2	Ref		6·0	**0·7**	**0·6, 0·8**	**4·5**	**0·4**	**0·3, 0·5**	**3·1**	1·1	0·9, 1·4	6·7
Child in the household															
No	Ref		65·1	Ref		84·1	Ref		71·3	Ref		56·6	Ref		79·1
Yes	**2·8**	**2·4, 3·3**	**34·9**	Ref		15·9	**2·1**	**1·8, 2·4**	**28·7**	**4·0**	**3·3, 5·0**	**43·4**	**1·4**	**1·2, 1·6**	**20·9**
Adolescent in the household															
No	Ref		92·2	Ref		89·0	Ref		90·9	Ref		92·3	Ref		87·5
Yes	**0·7**	**0·6, 0·8**	7·8	Ref		11·0	**0·8**	**0·7, 0·9**	9·1	**0·7**	**0·5, 0·8**	7·7	1·1	1·0, 1·4	12·5
**Anthropometric characteristics**															
BMI															
Under 18·5 kg/m²	**1·7**	**1·5, 2·1**	**7·7**	Ref		4·1	1·0	0·9, 1·2	3·9	1·0	0·8, 1·3	3·2	**1·7**	**1·4, 2·0**	**5·4**
18·5–25 kg/m²	Ref		65·1	Ref		60·4	Ref		54·8	Ref		48·1	Ref		46·5
25–30 kg/m²	**0·7**	**0·7, 0·8**	**20·9**	Ref		25·9	1·1	1·0, 1·2	26·4	**1·5**	**1·3, 1·7**	**31·1**	**1·5**	**1·4, 1·6**	**29·6**
Over 30 kg/m²	**0·6**	**0·5, 0·7**	**6·3**	Ref		9·6	**1·7**	**1·5, 1·9**	**14·9**	**2·3**	**2·0, 2·6**	**17·5**	**2·5**	**2·2, 2·8**	**18·5**

CU, consumption unit; UPF, ultra-processed foods.

Note. Pattern 2 was used as a reference in the model. Results for which 95 % CI excludes the null are bolded.

Example of interpretation: Female participants were 20 % more likely to be in the ‘Against all food taxes’ pattern and 10 % less likely to be in the ‘Support all food taxes’ pattern than in the ‘Support all but meat and fatty products taxes’ pattern.

* OR and 95 % CI were obtained using a multivariable logistic regression model.

† Refers to adjusted row percentage.

**Table 4.** Associations between participants’ diet and identified patterns: adjusted analyses (weighted sample) (*n* 15 862^‡^)

	Pattern 1			Pattern 2			Pattern 3			Pattern 4			Pattern 5		
	Support all food taxes			Support all but meat and fatty products taxes			Against all but UPF, Nutri-Score and salty products taxes			Against all food taxes			No opinion		
	*n* 2155, 13·6 %			*n* 4976, 31·4 %			*n* 4754, 30·0 %			*n* 1321, 8·3 %			*n* 2656, 16·7 %		
	OR*	95 % CI	%^†^	OR*	95 % CI	%^†^	OR*	95 % CI	%^†^	OR*	95 % CI	%^†^	OR*	95 % CI	%^†^
Fatty products															
≤ median	Ref		52·6	Ref		47·8	Ref		50·9	Ref		42·2	Ref		53·5
> median	**0·8**	**0·7, 0·9**	**47·4**	Ref		52·2	**0·9**	**0·8, 1·0**	**49·1**	**1·3**	**1·1, 1·4**	57·8	**0·8**	**0·7, 0·9**	46·5
Sugary products															
≤ median	Ref		50·9	Ref		51·7	Ref		50·9	Ref		44·3	Ref		47·6
> median	1·0	0·9, 1·2	49·1	Ref		48·3	1·0	0·9, 1·1	49·1	**1·3**	**1·2, 1·5**	55·7	**1·2**	**1·1, 1·3**	52·4
Salty products															
≤ median	Ref		48·3	Ref		57·1	Ref		55·4	Ref		34·9	Ref		51·1
> median	**1·4**	**1·3, 1·6**	**51·7**	Ref		42·9	**1·6**	**1·5, 1·8**	**44·6**	**2·5**	**2·1, 2·9**	65·1	**1·3**	**1·1, 1·4**	48·9
Fatty and sugary products															
≤ median	Ref		44·1	Ref		47·3	Ref		59·4	Ref		48·6	Ref		52·9
> median	**1·1**	**1·0, 1·2**	**55·9**	Ref		52·7	**0·8**	**0·8, 0·9**	40·6	0·9	0·8, 1·1	51·4	**0·8**	**0·7, 0·9**	47·1
Fatty and salty products															
≤ median	Ref		48·1	Ref		48·9	Ref		50·4	Ref		53·5	Ref		48·2
> median	1·0	0·9, 1·2	51·9	Ref		51·1	**0·9**	**0·8, 1·0**	**49·6**	**0·8**	**0·7, 0·9**	46·5	1·0	0·9, 1·1	51·8
Meat															
≤ median	Ref		67·7	Ref		46·9	Ref		46·7	Ref		45·1	Ref		53·2
> median	**0·4**	**0·4, 0·5**	**32·3**	Ref		53·1	1·0	0·9, 1·1	53·3	1·1	0·9, 1·2	54·9	**0·7**	**0·6, 0·8**	46·8
Products with an unfavourable Nutri-Score labelling															
≤ median	Ref		49·0	Ref		51·7	Ref		49·0	Ref		47·7	Ref		51·7
> median	1·1	1·0, 1·3	51·0	Ref		48·3	**1·2**	**1·1, 1·3**	**51·0**	**1·2**	**1·0, 1·4**	**52·3**	1·0	0·9, 1·1	48·3
Ultra-processed foods															
≤ median	Ref		60·2	Ref		53·6	Ref		47·5	Ref		44·0	Ref		46·7
> median	**0·8**	**0·7, 0·9**	**39·8**	Ref		46·4	**1·3**	**1·2, 1·4**	**52·5**	**1·5**	**1·3, 1·7**	**56·0**	**1·3**	**1·2, 1·5**	**53·3**

UPF, ultra-processed foods.

Note. Pattern 2 was used as a reference in the model. Results for which 95 % CI excludes the null are bolded.

Example of interpretation: Participants who consumed ultra-processed foods above the median were 50 % more likely to be in the ‘Against all food taxes’ pattern and 20 % less likely to be in the ‘Support all food taxes’ pattern than in the ‘Support all but meat and fatty products taxes’ pattern.

* OR and 95 % CI were obtained using a multivariable logistic regression model.

† Refers to adjusted row percentage.

‡ Data on dietary intake were available for 15 862 participants. This subsample was weighted on sex, age, education level, occupation, region of residence and marital status according to the 2016 National Census.

## Discussion

This study documented, in a large sample of the French adult population, that the acceptability of eight hypothetical food taxes varied according to the type of food subject to the tax. Specifically, taxes on UPF, salty products and products with an unfavourable Nutri-Score labelling were the most accepted, and taxes on meat and fatty products (including butter and cream) were the least accepted. Five patterns of food taxes acceptability were identified, highlighting that 8·6 and 16·9 % of participants were either against or in favour of most food taxes, respectively. Patterns of food taxes acceptability differed according to sociodemographic and anthropometric characteristics and dietary intake.

Participants in favour of taxes on meat and fatty products did so mainly for health and sustainability reasons. These reasons given by participants may be related to scientific evidence^(31)^. In addition, Godfray *et al.* showed that meat production, especially red meat, accounts for more than half of all greenhouse gases, contributing to climate change^(32)^, which is consistent with the accounting of livestock emissions of all greenhouse gases (at least 16·5 %)^(33)^. In the present study, we examined the acceptability of meat in general, not specifically red or processed meat (which was rather included as an example of fatty and salty products). Participants may be more willing to accept a tax on red meat than on poultry, given their different environmental and health impacts^(6,31,32)^. In this way, in addition to the potentially beneficial health effects of taxing unhealthy foods, such taxes could have beneficial side effects such as reducing our environmental footprint and helping to ‘green’ our diets^(32)^. There are several reasons for the overall low acceptability of meat taxes. Meat consumption is influenced by beliefs and social and cultural dimensions^(34)^, which translate into different motives for consuming (or not consuming) meat^(35)^. Accordingly, with existing theory, our results reflect that meat consumption is viewed as necessary by participants, as they most often cited nutritional quality as a reason for being against a meat tax^(36)^. In addition to socio-cultural dimensions, the nutritional properties of meat could explain the high proportion of participants against meat tax^(37)^, as meat is a rich source of protein and iron^(37)^.

The literature stated that the extent to which food taxes is likely to be acceptable matters because levels of acceptability may critically affect the effectiveness of such taxes in changing behaviour^(17,38)^. For example, participants in the ‘*Against all food taxes*’ pattern could be less likely to change their dietary intake than participants in other patterns, given their low acceptability of all food taxes. However, participants in the ‘*Against all food taxes*’ pattern appeared to belong to population subgroups more likely to have poorer diet quality (e.g. consumed the highest quantity of products with an unfavourable Nutri-Score labelling and UPF) and thus more prone to further health problems. In addition, proportions of participants with a BMI > 30 kg/m² (i.e. a proxy for dietary intake in our study) and without a high school diploma were highest in this pattern compared with other patterns, which are characteristics correlated with poor diet quality^(39)^. While improving dietary intake may be most needed among these participants, they are the ones for whom food taxes may be least effective. This underlines that the acceptability of food taxes may be explained by lifestyle habits^(34)^. Participants might be against food taxes because the foods that would be taxed and lead to price increases are the foods they usually buy and consume.

The implementation of taxes on unhealthy food raises the question of its regressivity (i.e. the tax burden could decrease with income) and its effect on social inequalities^(40)^. Specifically, such taxes could disproportionately affect people with the lowest incomes, especially since they are the ones who consume most of the taxed products^(10,17)^. If people with the lowest incomes continue to consume unhealthy products even after a tax on them is implemented, the result could be a worsening of the gap in food budgets between those with lower and higher incomes. However, the results of a systematic review suggest that the regressivity of a food tax (a tax on sugar-sweetened beverages) could be relatively low^(41)^. A recent umbrella review also suggests that taxes on unhealthy foods may have a positive (i.e. reduction) or neutral effect on socio-economic inequalities in diet quality, with greater price elasticity reported in lower socio-economic groups^(42)^. Drawing a parallel with tobacco taxes, where the long-term data are more abundant than for food taxes, the literature suggests that tobacco taxes could reduce social inequalities in smoking^(43)^.

The high number of participants with no opinion on food taxes (from 13·7 to 35·6 %) suggests that a relatively important part of the population would not be interested in debating such taxes. This could have policy implications, particularly with regard to the perceived risk that policymakers take in implementing a food tax and the communication that should accompany the implementation of food taxes. Given the large acceptability of a tax on UPF by the public (78·0 % in favour), it could be less risky for the government to implement such a tax, and there would be a definite benefit in raising the issue of the UPF tax in the public debate. Notably, in France, mass media coverage and a parliamentary commission of inquiry into the deleterious health effects of UPF, in part prompted by scientific research, may contribute to the greater acceptability of the UPF tax than other food taxes. The results regarding meat underline the ambiguity between participants who support and those who oppose a meat food tax on health grounds^(44)^. It suggests more communication (the optimal modalities of which have yet to be fully explored^(45)^) to sensitise the population on and influence their behaviour beyond the ‘price effect’ that would be generated by taxes, especially the socially less advantaged part of the population. Considering the Nutri-Score front-of-pack nutrition label, its overall relatively high acceptability by participants (54·6 % in favour) and the strong evidence of its beneficial health effects^(46)^, it would be appropriate and strategic to implement a food tax based on the Nutri-Score^(22)^. However, the current context in France, in particular with price inflation, may limit the implementation of such taxes. A combination of taxes on unhealthy foods and subsidies for other healthy and eco-friendly foods such as legumes, fruits and vegetables could be justified, increase public acceptability and limit potential inequalities^(10,47)^.

In addition to public acceptance of taxes on meat (and on other food products), the feasibility of such taxes could be limited by lobbying activities^(48)^. Big food companies could influence policy and governance through their lobbying activities, in order to increase their power and influence and to maintain a favourable regulatory environment for their products^(48)^. For example, a tax on processed meat in the USA may not be politically feasible, although legally possible, because of the considerable political power of the processed meat industry through its lobbies^(49)^. Another concrete example is the world’s first tax on saturated fats (including meat), introduced in Denmark in 2011^(50)^. The food industry lobby contributed to its repeal 15 months later using tactics such as threatening lawsuits, predicting welfare losses and questioning the evidence^(50)^.

The findings of this study should be interpreted in the light of its limitations and strengths. First, the participants in the study (crude sample) had a higher level of education and higher income and were older than the general French adult population, which may limit the representativeness and generalisability of the results to other non-French populations. However, we weighted the study sample to limit this bias and the characteristics of the weighted sample approached those of the general French population in terms of sociodemographic characteristics. Second, participants were asked to complete an initial questionnaire on sugar taxation – with items on the potential positive aspects of taxes – before answering the questionnaire in this study^(19)^. This may have had a priming effect on participants’ acceptability of taxes in general, but the identification of the ‘against all food taxes’ pattern suggests that this bias was rather limited in magnitude. Third, as the questionnaire was sent to participants before the period of high inflation following COVID-19, extrapolation of the results to the current period is uncertain. It is possible that people’s reactions to different taxes may be more critical in the context of an economic crisis. Fourth, it would have been interesting to examine the acceptability of food taxes at the same time as subsidies (e.g. for vegetables), but the questionnaire in our study only covered food taxes. Fifth, there was no example or definition of UPF in the questionnaire, but the latter aimed to measure the spontaneous reaction to a possible tax on UPF among the public regardless of their level of literacy on the subject. In addition, our team’s research showing associations with disease risk (e.g. CVD and type 2 diabetes^(4)^) has helped to make the concept of UPF well known and highly visible to the general public. It is therefore likely that the general public has a relatively accurate idea of the overall definition of the UPF concept. Lastly, the cross-sectional design did not allow for causal inferences. Strengths include the national and large study sample, the variety of potential food taxes examined and the combination of both acceptability and reasons for acceptability of food taxes in the analyses.

### Conclusions

This study evidenced that among eight hypothetical food taxes, participants were most in favour of taxes on UPF, salty products and products with an unfavourable Nutri-Score labelling and most against taxes on meat and fatty products (added fats) in a large sample of French adults. Five patterns of food taxes acceptability were identified, with a total of 8·6 and 16·9 % of the participants belonging to the patterns against and in favour of all food taxes, respectively. Participants against all food taxes were more likely to be from lower socio-economic status, with a high BMI and with poor diet quality than participants in the other patterns. The findings will be of interest to policymakers in the design of food taxes and suggest further research to identify levers that could improve the acceptability of all food taxes across the adult population, especially those from lower socio-economic status.

## Supporting information

Manneville et al. supplementary material 1 Manneville et al. supplementary material

Manneville et al. supplementary material 2 Manneville et al. supplementary material

## Supplementary material

For supplementary material accompanying this paper, visit https://doi.org/10.1017/S1368980024002556.
