# Supplementary material for: Acceptability patterns of hypothetic taxes on different types of foods in France
Source: Public Health Nutr. 2024 Dec 26;28(1):e18. doi: 10.1017/S1368980024002556 (PMC11822589; doi:10.1017/S1368980024002556)
Supplement: Manneville et al. supplementary material 2 — Manneville et al. supplementary material [file S1368980024002556sup002.docx]

**Supplementary Material**

**Table S1.** Description of participants’ dietary intake (weighted sample) (N= 15,862^a^)

|  | Median | Q1-Q3 |
| --- | --- | --- |
| **Fatty products** (g/day) | 11.0 | 5.4-19.8 |
| **Sugary products** (g/day) | 49.8 | 27.6-82.8 |
| **Salty products** (g/day) | 140.9 | 87.2-201.6 |
| **Fatty and sugary products** (g/day) | 58.3 | 32.2-92.2 |
| **Fatty and salty products** (g/day) | 49.4 | 32.0-72.9 |
| **Meat** (g/day) | 42.6 | 26.1-65.9 |
| **Products with an unfavorable Nutri-Score labeling** (g/day) | 197.2 | 142.5-280.1 |
| **Ultra-processed foods** (g/day) | 346.8 | 259.4-459.4 |

*^a^ data on dietary intake were available for15,862 participants. This subsample was weighted on sex, age, education level, occupation, region of residence, and marital status according to the 2016 National Census.*

**Table S2.** Description of model fit statistics, pattern proportions, and posterior probabilities of the latent class analysis process

|  | **Two-pattern**  **model** | **Three-pattern**  **model** | **Four-pattern**  **model** | **Five-pattern**  **model** | **Six-pattern**  **model** |
| --- | --- | --- | --- | --- | --- |
| **Akaike information criterion** | 935196 | 892865 | 864865 | 839164 | 821664 |
| **Bayesian information criterion** | 937708 | 897096 | 869898 | 845457 | 829217 |
| **Entropy** | 0.90 | 0.91 | 0.89 | 0.89 | 0.89 |
| **Pattern size (proportions^a^). %** |  |  |  |  |  |
| Pattern 1 | 13240 (0.47) | 11281 (0.40) | 4441 (0.16) | 4728 (0.17) | 2481 (0.09) |
| Pattern 2 | 14660 (0.53) | 3582 (0.13) | 8431 (0.30) | 8050 (0.29) | 7836 (0.28) |
| Pattern 3 | - | 13037 (0.47) | 12144 (0.44) | 7384 (0.26) | 3446 (0.12) |
| Pattern 4 | - | - | 2884 (0.10) | 2405 (0.09) | 4250 (0.15) |
| Pattern 5 | - | - | - | 5333 (0.19) | 3744 (0.14) |
| Pattern 6 | - | - | - | - | 6143 (0.22) |
| **Posterior probabilities^a^. %** |  |  |  |  |  |
| Pattern 1 | 0.98 | 0.97 | 0.93 | 0.96 | 0.97 |
| Pattern 2 | 0.97 | 0.96 | 0.94 | 0.94 | 0.92 |
| Pattern 3 | - | 0.96 | 0.95 | 0.94 | 0.94 |
| Pattern 4 | - | - | 0.97 | 0.97 | 0.97 |
| Pattern 5 | - | - | - | 0.94 | 0.94 |
| Pattern 6 | - | - | - | - | 0.94 |

*^a^ The sum of the proportions could be different from 0 due to rounding.*

*Note: Five latent class models were computed to obtain model fit statistics, pattern proportions, and posterior probabilities.*

**Table S3.** Distribution (%) acceptability for food taxes according to identified patterns of food taxes acceptability (weight sample) (N=27,900)

|  |  | Pattern 1  Support all food taxes  (n= 4,728, 16.9%) | Pattern 2  Support all but meat and fatty products taxes  (n= 8,050, 28.9%) | Pattern 3  Against all but UPF, Nutri-score, and salty products taxes  (n= 7,384, 26.5%) | Pattern 4  Against all food taxes  (n= 2,405, 8.6%) | Pattern 5  No opinion  (n= 5,333, 19.1%) |
| --- | --- | --- | --- | --- | --- | --- |
| **Fatty products** | Strongly disagree | 8.9 | 22.3 | 53.3 | 73.9 | 11.1 |
|  | Somewhat disagree | 18.9 | 39.5 | 36.5 | 18.3 | 10.8 |
|  | Neither agree nor disagree | 27.0 | 29.6 | 9.6 | 2.9 | 73.6 |
|  | Somewhat agree | 19.6 | 6.5 | 0.5 | 1.1 | 2.1 |
|  | Strongly agree | 25.6 | 2.0 | 0.1 | 3.8 | 2.4 |
| **Sugary products** | Strongly disagree | 0.3 | 0.2 | 14.1 | 56.5 | 2.7 |
|  | Somewhat disagree | 1.5 | 1.8 | 30.7 | 32.4 | 5.9 |
|  | Neither agree nor disagree | 14.0 | 16.3 | 33.0 | 10.2 | 81.7 |
|  | Somewhat agree | 51.4 | 53.1 | 18.2 | 0.7 | 7.2 |
|  | Strongly agree | 32.8 | 28.6 | 3.9 | 0.2 | 2.5 |
| **Salty products** | Strongly disagree | 0.6 | 0.6 | 4.9 | 54.3 | 1.7 |
|  | Somewhat disagree | 2.1 | 2.2 | 15.2 | 33.3 | 4.2 |
|  | Neither agree nor disagree | 8.6 | 9.3 | 36.9 | 12.1 | 76.6 |
|  | Somewhat agree | 44.4 | 56.2 | 31.5 | 0.3 | 13.6 |
|  | Strongly agree | 44.4 | 31.8 | 11.5 | 0.1 | 3.8 |
| **Fatty and sugary products** | Strongly disagree | 0.2 | 0.1 | 14.3 | 60.3 | 2.4 |
|  | Somewhat disagree | 3.9 | 1.1 | 40.2 | 32 | 3.1 |
|  | Neither agree nor disagree | 18.6 | 24.4 | 36.0 | 7.0 | 84.6 |
|  | Somewhat agree | 46.8 | 53.1 | 8.3 | 0.4 | 8.6 |
|  | Strongly agree | 30.4 | 24.3 | 1.2 | 0.4 | 1.3 |
| **Fatty and salty products** | Strongly disagree | 0.3 | 3.2 | 23.1 | 61.0 | 3.8 |
|  | Somewhat disagree | 4.1 | 13.8 | 40.2 | 31.6 | 4.4 |
|  | Neither agree nor disagree | 15.4 | 29.8 | 29.1 | 3.1 | 82.1 |
|  | Somewhat agree | 38.5 | 38.5 | 6.5 | 0.5 | 9.1 |
|  | Strongly agree | 41.7 | 14.7 | 1.2 | 3.8 | 0.6 |
| **Meat** | Strongly disagree | 0.5 | 30.6 | 54.7 | 71.2 | 14.1 |
|  | Somewhat disagree | 1.1 | 38.3 | 31.3 | 17.6 | 14.9 |
|  | Neither agree nor disagree | 17.3 | 30.6 | 8.5 | 3.6 | 59.1 |
|  | Somewhat agree | 40.9 | 0.4 | 4.3 | 2.1 | 7.5 |
|  | Strongly agree | 40.2 | 0.0 | 1.2 | 5.6 | 4.4 |
| **Products with an unfavorable Nutri-Score labeling** | Strongly disagree | 1.7 | 1.3 | 9.2 | 48.2 | 3.9 |
|  | Somewhat disagree | 5.1 | 3.8 | 15.2 | 33.2 | 4.8 |
|  | Neither agree nor disagree | 12.3 | 14.7 | 28.1 | 14.0 | 66.0 |
|  | Somewhat agree | 35.1 | 47.1 | 32.2 | 3.5 | 18.1 |
|  | Strongly agree | 45.9 | 33.1 | 15.3 | 1.1 | 7.2 |
| **Ultra-processed foods** | Strongly disagree | 0.4 | 0.6 | 1.4 | 39.6 | 3.0 |
|  | Somewhat disagree | 0.4 | 0.2 | 2.4 | 26.4 | 3.0 |
|  | Neither agree nor disagree | 2.9 | 1.7 | 10.6 | 17.1 | 44.4 |
|  | Somewhat agree | 24.9 | 30.4 | 45.5 | 12.1 | 28.7 |
|  | Strongly agree | 71.3 | 67.1 | 40.1 | 4.9 | 20.9 |

*Note: the data presented in this table are the data used to create Figure 2.*

**Table S4.** Associations between participants’ sociodemographic and anthropometric characteristics and identified patterns: unadjusted analyses (weighted sample) (N =27,900)

|  | Pattern 1  Support all food taxes  (n= 4,728, 16.9%) | | | Pattern 2  Support all but meat and fatty products taxes  (n= 8,050, 28.9%) | | | Pattern 3  Against all but UPF, Nutri-score, and salty products taxes  (n=7,384, 26.5%) | | | Pattern 4  Against all food taxes  (n= 2,405, 8.6%) | | | Pattern 5  No opinion  (n= 5,333, 19.1%) | | |
| --- | --- | --- | --- | --- | --- | --- | --- | --- | --- | --- | --- | --- | --- | --- | --- |
|  | OR^a^ | 95% CI | %^b^ | OR^a^ | 95% CI | %^b^ | OR^a^ | 95% CI | %^b^ | OR^a^ | 95% CI | %^b^ | OR^a^ | 95% CI | %^b^ |
| **Sociodemographic characteristics** |  |  |  |  |  |  |  |  |  |  |  |  |  |  |  |
| Sex |  |  |  |  |  |  |  |  |  |  |  |  |  |  |  |
| Male | Ref |  | 50.5 | Ref |  | 48.0 | Ref |  | 44.4 | Ref |  | 41.7 | Ref |  | 51.6 |
| Female | **0.9** | **0.8-1.0** | **49.5** | Ref |  | 52.0 | **1.2** | **1.1-1.2** | **55.6** | **1.3** | **1.2-1.4** | **58.3** | **0.9** | **0.8-0.9** | **48.4** |
| Age |  |  |  |  |  |  |  |  |  |  |  |  |  |  |  |
| 18-30 years | **1.7** | **1.5-1.9** | **23.4** | Ref |  | 12.1 | 1.0 | 0.9-1.1 | 13.6 | **1.3** | **1.1-1.5** | **18.0** | **1.3** | **1.1-1.4** | **14.6** |
| 30-44 years | Ref |  | 23.2 |  |  | 20.5 | Ref |  | 23.0 | Ref |  | 19.7 | Ref |  | 23.3 |
| 45-54 years | **0.8** | **0.7-0.9** | **26.5** | Ref |  | 28.8 | **0.9** | **0.8-1.0** | **29.5** | 1.0 | 0.9-1.2 | 33.8 | 1.1 | 1.0-1.2 | 29.6 |
| 55-65 years | **0.5** | **0.5-0.6** | **11.9** | Ref |  | 19.8 | **0.8** | **0.7-0.9** | **17.8** | **0.6** | **0.5-0.7** | **13.0** | **0.8** | **0.7-0.9** | **14.9** |
| Over 65 years | **0.7** | **0.6-0.8** | **14.9** | Ref |  | 18.7 | **0.8** | **0.7-0.8** | **16.1** | **0.6** | **0.5-0.7** | **11.9** | **1.2** | **1.1-1.3** | **21.2** |
| Income |  |  |  |  |  |  |  |  |  |  |  |  |  |  |  |
| Less than €1300/mont/CU | **1.2** | **1.1-1.3** | **28.5** | Ref |  | 22.7 | **0.8** | **0.7-0.8** | **21.5** | **0.8** | **0.7-0.9** | **21.6** | **0.8** | **0.7-0.9** | **20.8** |
| €1300-2600/month/CU | Ref |  | 41.4 | Ref |  | 38.7 | Ref |  | 46.9 | Ref |  | 44.2 | Ref |  | 45.2 |
| Over €2600/month/CU | **0.7** | **0.6-0.8** | **20.0** | Ref |  | 26.1 | **0.6** | **0.5-0.6** | **18.4** | **0.6** | **0.5-0.7** | **18.4** | **0.5** | **0.4-0.5** | **15.0** |
| Did not want to declare | **0.8** | **0.7-0.9** | **10.2** | Ref |  | 12.4 | **0.9** | **0.8-1.0** | **13.3** | 1.1 | 1.0-1.3 | 15.8 | **1.3** | **1.2-1.4** | **18.9** |
| Education |  |  |  |  |  |  |  |  |  |  |  |  |  |  |  |
| No high school diploma | 0.9 | 0.8-1.1 | 7.3 | Ref |  | 8.3 | 1.1 | 0.9-1.2 | 10.1 | **1.3** | **1.1-1.6** | **12.0** | **1.5** | **1.3-1.7** | **15.0** |
| High school diploma | Ref |  | 37.0 |  |  | 40.3 | Ref |  | 46.0 | Ref |  | 43.0 | Ref |  | 47.9 |
| University degree | **1.2** | **1.1-1.3** | **55.6** | Ref |  | 51.4 | **0.7** | **0.7-0.8** | **43.8** | **0.8** | **0.7-0.9** | **45.0** | **0.6** | **0.6-0.7** | **37.1** |
| Occupation |  |  |  |  |  |  |  |  |  |  |  |  |  |  |  |
| Farmer | **0.1** | **0.0-0.2** | **0.1** | Ref |  | 0.9 | **2.0** | **1.5-2.6** | **1.8** | **0.2** | **0.1-0.6** | **0.2** | **1.7** | **1.2-2.5** | **0.9** |
| Artisan, Shopkeeper, Business owner | 0.9 | 0.7-1.1 | 3.9 | Ref |  | 3.7 | 1.0 | 0.8-1.2 | 3.5 | 1.1 | 0.8-1.4 | 3.4 | **2.3** | **1.9-2.8** | **4.9** |
| Manager of higher intellectual profession | Ref |  | 16.2 | Ref |  | 13.9 | Ref |  | 13.7 | Ref |  | 11.6 | Ref |  | 8.0 |
| Intermediate profession | **0.6** | **0.5-0.6** | **9.9** | Ref |  | 15.0 | 0.7 | 0.6-0.8 | 9.9 | **0.8** | **0.7-1.0** | **10.1** | **0.6** | **0.5-0.7** | **5.1** |
| Employee | **0.7** | **0.6-0.8** | **13.9** | Ref |  | 16.4 | 1.2 | 1.1-1.3 | 19.4 | **1.9** | **1.6-2.2** | **25.6** | **2.0** | **1.7-2.3** | **18.9** |
| Worker | **0.9** | **0.8-1.0** | **10.5** | Ref |  | 10.1 | 0.9 | 0.8-1.1 | 9.4 | **0.6** | **0.5-0.8** | **5.2** | **2.6** | **2.2-3.0** | **14.8** |
| Student | **1.2** | **1.0-1.4** | **9.4** | Ref |  | 6.8 | **0.7** | **0.6-0.8** | **4.8** | **0.8** | **0.6-0.8** | **4.4** | **2.3** | **2.0-2.7** | **9.0** |
| Retired person | **0.7** | **0.6-0.8** | **12.9** | Ref |  | 14.8 | **1.1** | **0.9-1.2** | **15.6** | 0.9 | 0.7-1.0 | 10.6 | **2.3** | **2.0-2.6** | **19.2** |
| No activity | 1.1 | 1.0-1.2 | 23.3 | Ref |  | 18.4 | **1.2** | **1.1-1.3** | **21.8** | **1.9** | **1.6-2.2** | **28.9** | **1.8** | **1.6-2.1** | **19.2** |
| Region of residence of France |  |  |  |  |  |  |  |  |  |  |  |  |  |  |  |
| North | 1.0 | 0.9-1.1 | **9.6** | Ref |  | 9.0 | **1.2** | **1.1-1.4** | **10.6** | 0.9 | 0.7-1.0 | 8.3 | 1.0 | 0.8-1.1 | 7.0 |
| Île-de-France | Ref |  | 20.0 | Ref |  | 18.8 | Ref |  | 17.9 | Ref |  | 20.2 | Ref |  | 15.1 |
| Paris basin | **0.7** | **0.6-0.8** | **7.5** | Ref |  | 9.9 | 1.1 | 0.9-1.2 | 10.1 | **0.8** | **0.7-1.0** | **8.6** | **1.2** | **1.1-1.4** | **9.8** |
| East Center | **0.7** | **0.6-0.8** | **9.0** | Ref |  | 11.8 | **1.1** | **1.0-1.3** | **12.8** | 1.2 | 1.0-1.4 | 14.6 | **1.2** | **1.0-1.3** | **11.2** |
| East | 1.0 | 0.9-1.1 | 14.1 | Ref |  | 13.0 | 1.0 | 0.9-1.1 | 12.1 | **0.7** | **0.5-0.8** | **9.2** | 1.1 | 0.9-1.3 | 11.5 |
| West | **0.8** | **0.7-1.0** | **9.6** | Ref |  | 10.8 | **1.1** | **1.0-1.3** | **11.8** | 0.9 | 0.8-1.1 | 10.6 | **1.4** | **1.2-1.6** | **11.8** |
| South West | 1.1 | 1.0-1.2 | 22.2 | Ref |  | 18.8 | 0.9 | 0.9-1.1 | 17.1 | 1.0 | 0.9-1.1 | 20.8 | **1.3** | **1.2-1.5** | **20.3** |
| Mediterranean | 0.9 | 0.8-1.1 | **8.0** | Ref |  | 7.9 | 1.0 | 0.9-1.1 | 7.5 | 0.9 | 0.7-1.1 | 7.7 | **2.1** | **1.8-2.4** | **13.2** |
| Marital status |  |  |  |  |  |  |  |  |  |  |  |  |  |  |  |
| Married | Ref |  | 36.2 | Ref |  | 52.8 | Ref |  | 48.7 | Ref |  | 41.9 | Ref |  | 43.2 |
| In a couple | **2.0** | **1.8-2.2** | **25.1** | Ref |  | 18.2 | **1.3** | **1.2-1.6** | **22.4** | **1.6** | **1.5-1.9** | **23.8** | **1.2** | **1.1-1.4** | **18.4** |
| Divorced or separated | **1.6** | **1.4-1.8** | **9.6** | Ref |  | 8.7 | 0.9 | 0.8-1.1 | 7.6 | **1.3** | **1.1-1.5** | **9.0** | 1.1 | 1.0-1.3 | 7.9 |
| Widow | **0.7** | **0.5-0.9** | **1.6** | Ref |  | 3.3 | 0.9 | 0.7-1.1 | 2.8 | **0.7** | **0.5-1.0** | **1.9** | **1.8** | **1.5-2.1** | **4.9** |
| Single | **2.4** | **2.1-2.6** | **27.5** | Ref |  | 17.0 | **1.2** | **1.1-1.3** | **18.5** | **1.7** | **1.5-2.0** | **23.4** | **1.8** | **1.7-2.0** | **25.6** |
| Number of persons in the household |  |  |  |  |  |  |  |  |  |  |  |  |  |  |  |
| 1 | 1.0 | 0.9-1.1 | 24.7 | Ref |  | 23.4 | 1.0 | 0.9-1.1 | 22.6 | 1.1 | 0.9-1.2 | 25.5 | **1.5** | **1.3-1.6** | **30.3** |
| 2 | Ref |  | 43.2 | Ref |  | 41.3 | Ref |  | 40.5 | Ref |  | 42.4 | Ref |  | 36.8 |
| 3 | **0.8** | **0.7-0.9** | **14.1** | Ref |  | 17.5 | 1.1 | 1.0-1.2 | 18.4 | 0.9 | 0.8-1.1 | 16.7 | 1.1 | 1.0-1.2 | 16.7 |
| 4 | **0.8** | **0.7-0.9** | **9.7** | Ref |  | 12.1 | **1.1** | **1.0-1.3** | **13.7** | 0.9 | 0.8-1.0 | 11.0 | 1.0 | 0.9-1.1 | 10.9 |
| >=5 | **1.4** | **1.2-1.6** | **8.3** | Ref |  | 5.7 | **0.8** | **0.7-1.0** | **4.8** | **0.7** | **0.6-0.9** | **4.3** | 1.0 | 0.9-1.2 | 5.2 |
| Child in the household |  |  |  |  |  |  |  |  |  |  |  |  |  |  |  |
| No | Ref |  | 73.8 | Ref |  | 79.8 | Ref |  | 72.9 | Ref |  | 72.0 | Ref |  | 78.1 |
| Yes | **1.4** | **1.3-1.5** | **26.2** | Ref |  | 20.2 | **1.5** | **1.4-1.6** | **27.1** | **1.5** | **1.4-1.7** | **28.0** | **1.1** | **1.0-1.2** | **21.9** |
| Adolescent in the household |  |  |  |  |  |  |  |  |  |  |  |  |  |  |  |
| No | Ref |  | 89.6 | Ref |  | 90.6 | Ref |  | 89.5 | Ref |  | 90.1 | Ref |  | 89.1 |
| Yes | 1.1 | 1.0-1.3 | 10.4 | Ref |  | 9.4 | **1.1** | **1.0-1.3** | **10.5** | 1.0 | 0.9-1.2 | 9.9 | **1.2** | **1.0-1.3** | **10.9** |
| **Anthropometric characteristic** |  |  |  |  |  |  |  |  |  |  |  |  |  |  |  |
| Body Mass Index |  |  |  |  |  |  |  |  |  |  |  |  |  |  |  |
| Under 18.5 kg/m² | **2.1** | **1.8-2.4** | **8.3** | Ref |  | 3.6 | 1.1 | 0.9-1.3 | 3.7 | **1.3** | **1.0-1.7** | **3.9** | **1.8** | **1.5-2.2** | **5.1** |
| 18.5-25kg/m² | 52.6 |  | 65.2 | Ref |  | 59.1 | Ref |  | 54.4 | 53.6 |  | 52.6 | 48.6 |  | 44.2 |
| 25-30 kg/m² | **0.7** | **0.6-0.7** | **25.2** | Ref |  | 28.0 | **1.1** | **1.0-1.2** | **27.9** | **1.3** | **1.2-1.4** | **25.7** | **1.5** | **1.4-1.6** | **35.8** |
| Over 30kg/m² | 0.6 | 0.5-0.6 | 9.2 | Ref |  | 9.3 | **1.8** | **1.6-1.9** | **14.9** | **2.2** | **1.9-2.5** | **17.5** | **2.4** | **2.2-2.7** | **17.0** |

*Abbreviations: OR, odds ratio; 95% CI, Confidence interval.*

*^a^OR and 95% CI were obtained using univariable logistic regression models*

*^b^refers to adjusted row percentage*

*Note. Pattern 2 was used as reference in the model. Results for which 95% CI excludes the null are bolded.*

**Table S5.** Associations between participants’ diet and identified patterns: unadjusted analyses (weighted sample) (N=15,862^c^)

|  | Pattern 1  Support all food taxes  (n= 2,155, 13.6%) | | | Pattern 2  Support all but meat and fatty products taxes  (n= 4,976, 31.4%) | | | Pattern 3  Against all but UPF, Nutri-score, and salty products taxes  (n= 4,754, 30.0%) | | | Pattern 4  Against all food taxes  (n= 1,321, 8.3%) | | | Pattern 5  No opinion  (n= 2,656, 16.7%) | | |
| --- | --- | --- | --- | --- | --- | --- | --- | --- | --- | --- | --- | --- | --- | --- | --- |
|  | OR^a^ | 95% CI | %^b^ | OR^a^ | 95% CI | %^b^ | OR^a^ | 95% CI | %^b^ | OR^a^ | 95% CI | %^b^ | OR^a^ | 95% CI | %^b^ |
| **Fatty products** |  |  |  |  |  |  |  |  |  |  |  |  |  |  |  |
| ≤median | Ref |  | 52.8 | Ref |  | 47.4 | Ref |  | 51.8 | Ref |  | 42.8 | Ref |  | 53.9 |
| >median | 0.8 | 0.7-0.9 | 47.2 | Ref |  | 52.6 | **0.8** | **0.8-0.9** | **48.2** | **1.2** | **1.1-1.4** | **57.2** | **0.8** | **0.7-0.8** | **46.1** |
| **Sugary products** |  |  |  |  |  |  |  |  |  |  |  |  |  |  |  |
| ≤median | Ref |  | 53.1 | Ref |  | 51.4 | Ref |  | 50.5 | Ref |  | 43.0 | Ref |  | 47.7 |
| >median | 0.9 | 0.8-1.0 | 46.9 | Ref |  | 48.6 | 1.0 | 1.0-1.1 | 49.5 | **1.4** | **1.2-1.6** | **57.0** | **1.2** | **1.1-1.3** | **52.3** |
| **Salty products** |  |  |  |  |  |  |  |  |  |  |  |  |  |  |  |
| ≤median | Ref |  | 47.9 | Ref |  | 58.1 | Ref |  | 44.4 | Ref |  | 36.7 | Ref |  | 53.1 |
| >median | **1.5** | **1.4-1.7** | **52.1** | Ref |  | 41.9 | **1.7** | **1.6-1.9** | **55.6** | **2.4** | **2.1-2.7** | **63.3** | **1.2** | **1.1-1.3** | **46.9** |
| **Fatty and sugary products** |  |  |  |  |  |  |  |  |  |  |  |  |  |  |  |
| ≤median | Ref |  | 44.3 | Ref |  | 50.1 | Ref |  | 50.9 | Ref |  | 44.3 | Ref |  | 53.9 |
| >median | **1.3** | **1.1-1.4** | **55.6** | Ref |  | 49.9 | 1.0 | 0.9-1.0 | 49.1 | **1.3** | **1.1-1.4** | **55.7** | **0.9** | **0.8-0.9** | **46.1** |
| **Fatty and salty products** |  |  |  |  |  |  |  |  |  |  |  |  |  |  |  |
| ≤median | Ref |  | 50.4 | Ref |  | 48.0 | Ref |  | 50.6 | Ref |  | 52.6 | Ref |  | 51.0 |
| >median | 0.9 | 0.8-1.0 | 49.6 | Ref |  | 52.0 | **0.9** | **0.8-1.0** | **49.4** | **0.8** | **0.7-0.9** | **47.4** | **0.9** | **0.8-1.0** | **49.0** |
| **Meat** |  |  |  |  |  |  |  |  |  |  |  |  |  |  |  |
| ≤median | Ref |  | 68.1 | Ref |  | 47.2 | Ref |  | 45.6 | Ref |  | 45.9 | Ref |  | 52.1 |
| >median | **0.4** | **0.4-0.5** | **31.8** | Ref |  | 52.8 | 1.1 | 1.0-1.1 | 54.4 | 1.0 | 0.9-1.2 | 54.1 | **0.8** | **0.7-0.9** | **47.9** |
| **Products with Nutri-Score D-E** |  |  |  |  |  |  |  |  |  |  |  |  |  |  |  |
| ≤median | Ref |  | 45.7 | Ref |  | 54.4 | Ref |  | 47.6 | Ref |  | 42.4 | Ref |  | 53.3 |
| >median | **1.4** | **1.3-1.6** | **54.3** | Ref |  | 45.6 | **1.3** | **1.2-1.4** | **52.4** | **1.6** | **1.4-1.8** | **57.6** | 1.0 | 0.9-1.1 | 46.7 |
| **Ultra-processed foods** |  |  |  |  |  |  |  |  |  |  |  |  |  |  |  |
| ≤median | Ref |  | 55.1 | Ref |  | 55.1 | Ref |  | 46.2 | Ref |  | 38.8 | Ref |  | 48.7 |
| >median | 1.0 | 0.9-1.1 | 44.9 | Ref |  | 44.9 | **1.4** | **1.3-1.5** | **53.8** | **1.9** | **1.7-2.2** | **61.2** | **1.3** | **1.2-1.4** | **51.3** |

*Abbreviations: OR, odds ratio; 95% CI, Confidence interval.*

*^a^OR and 95% CI were obtained using univariable logistic regression models*

*^b^refers to adjusted row percentage*

*^c^data on dietary intake were available for15,862 participants. This subsample was weighted on sex, age, education level, occupation, region of residence, and marital status* *according to the 2016 National Census.*

*Note. Pattern 2 was used as reference in the model. Results for which 95% CI excludes the null are bolded*
